# Self-reported physical health among people who switched from traditional cigarettes to heated tobacco products: a large-scale national survey

**DOI:** 10.1186/s12954-026-01466-2

**Published:** 2026-05-06

**Authors:** Agnieszka Tuszyńska-Chyży, Magdalena Walicka, Ludomir Tuszyński, Aleksandra Gola-Graczyk, Aleksandra Pohadajło, Iwona Towpik, Dorota Kiedik, Andrzej Fal, Edward Franek, Janusz Sytnik-Czetwertyński

**Affiliations:** 1 Clinical Department of Internal Medicine, Endocrinology and Diabetology, National Medical Institute of the Ministry of the Interior and Administration, Warsaw, Poland; 2 Mossakowski Medical Research Institute, Polish Academy of Sciences, Ul. Pawińskiego 5, 02-106 Warszawa, Poland; 3 Faculty of Electrical Engineering, Automatic Control and Computer Science, Kielce University of Technology, Kielce, Poland; 4 School of Public Health, Centre of Postgraduate Medical Education, Warsaw, Poland; 5 Department of Internal Medicine, University of Zielona Góra, Zielona Góra, Poland; 6 Department of Public Health, Division of Public Health, Faculty of Health Sciences, Wroclaw Medical University, Wroclaw, Poland; 7 Faculty of Medicine, Collegium Medicum, Cardinal Stefan Wyszyński University in Warsaw, Warsaw, Poland

**Keywords:** Heated tobacco products, Traditional cigarettes, Harm, Health effects

## Abstract

**Background:**

Traditional cigarettes (TC) are regarded as more hazardous compared to heated tobacco products (HTPs). However, mainly from ethical reasons, there are no well-designed studies examining the health impact of exposure to HTPs. This study aimed to assess and compare self-reported physical health implications of respondents smoking traditional cigarettes and HTPs.

**Methods:**

The survey was conducted in 2,500 adults who had smoked TC for at least a year and after quitting them had been using HTPs for a minimum of six months. The questions, between other issues, aimed to determine and compare the opinion of respondents on impact of smoking cigarettes and using HTPs on physical health, physical condition, and physical health symptoms.

**Results:**

Comparing TC and HTPs, 54.2% of the respondents indicated that TC were worse for their physical health, in turn 5.2% of respondents pointed to HTPs as worse. Similarly, 61.9% of respondents viewed TCs as more harmful to their physical condition, while 25% believed that more harmful are HTPs. Traditional cigarettes had reportedly more pronounced negative impact on various physical signs and symptoms.

**Conclusions:**

Most people who switched from traditional cigarettes to HTPs perceive traditional cigarettes as more harmful to their physical health. Most believe also that traditional cigarettes more negatively affect physical endurance and various physical signs and symptoms. Age appears to be the main demographic factor influencing perceptions, while other variables such as sex, income, and education have only minor effects.

**Supplementary Information:**

The online version contains supplementary material available at 10.1186/s12954-026-01466-2.

## Introduction

In recent years, the worldwide rate of tobacco smoking has declined [1]; however novel products containing tobacco or nicotine have entered the market, including electronic cigarettes (vaporizers) and heated tobacco products (HTPs). The use of e-cigarettes involves inhaling a vapor from a nicotine-containing liquid, whereas HTPs deliver nicotine by heating the tobacco, instead of combusting it. Between 2018 and 2020, there was a noticeable rise in the retail sales volumes of HTPs in EU Member States. The retail sales value of HTPs corresponded to 3.51% of the total sales volume of all tobacco products at the EU level in 2020 [2]. The use of HTPs is more common among younger people as well as among individuals who formerly or currently smoke, and the most common reason for use is the perception that they are less harmful than other tobacco products [3].

While traditional cigarettes have long been associated with severe health risks, including cardiovascular disease, respiratory disorders, and various cancers, HTPs are often marketed as a potentially safer alternative. However, the relative safety of HTPs remains a subject of debate, with emerging research indicating that these products, although less harmful in some respects, still pose significant health risks. While individuals using HTPs may be exposed to lower levels of certain toxicants like tobacco-specific nitrosamines, tar, carbon monoxide, aromatic amines, hydrogen cyanide, ammonia, phenol, volatile organic compounds, polycyclic aromatic hydrocarbons, carbonyls compared to traditional cigarettes, they may also be exposed to similar or even higher levels of other toxicants (hexadecanoic acid, ethyl ester, stearate, ethyl-, butylated hydroxytoluen, ethyl linoleate, propylene glycol, 2-furanmethanol, butyrolactone, methyl furoate, glycidol, ethyl linolenate are example constituents reported to be more than two-fold higher in the mainstream aerosol of HTPs compared with the mainstream smoke of traditional combustible cigarettes [4], potentially leading to adverse health effects.

HTPs, when compared to traditional cigarettes, may deliver varying levels of nicotine in their mainstream emissions depending on the specific device and brand. While some HTPs have been reported to deliver less nicotine, others can provide comparable or even higher amounts, especially as device technology advances. Moreover, improvements such as enhanced battery capacity and appealing taste (flavoured HTPs), might actually lead to increased consumption rather than reduced use. It should be noted that the impact of using HTPs on health remains uncertain, especially when considering long-term use. The existing toxicity data for HTPs primarily focus on their effects on the respiratory and cardiovascular systems [5, 6]. Independent studies reported a mix of outcomes, including beneficial, harmful, or similar effects of HTPs compared to conventional smoking [7, 8, 9, 10, 11]. However, there are no well-designed, long-term epidemiological studies examining the health impacts of exposure to HTPs yet, and it should be noted that much data on HTPs health outcomes comes from tobacco industry-sponsored studies [12]. Additionally, the design of interventional clinical trials has been limited by ethical concerns; for this and other reasons, the execution and documentation of HTP-related trials have often been inadequate and primarily focused on short-term exposure [13].

Therefore, there is still limited independent evidence directly comparing the health effects of HTPs and traditional cigarettes. To address this gap, we conducted a survey study evaluating self-reported physical health outcomes among individuals with firsthand experience of both cigarette smoking and HTP use.

## Materials and methods

The study was conducted in cooperation with the Public Opinion Research Center (CBOS) in Poland, which played an integral role in designing the primary research instrument—the survey questionnaire—and developing its electronic version for respondent use. Upon completion of this phase, CBOS delegated the implementation of the survey, which was carried out using the CAWI (Computer—Assisted Web Interview) method. The research was conducted by two independent institutes: IQS Think Forward sp. z o.o., a leader in online market research in Poland, and Pollster, a specialist in research using advanced technologies. These institutes were responsible for administering the electronic survey gathering the final data, and submitting it to CBOS as the lead partner. A detailed description of the methodology can be found in the supplementary material. In brief, the survey included 2,500 anonymous adult respondents who independently completed an electronic questionnaire. Participation in the survey was voluntary. Study participants had smoked traditional cigarettes for at least a year and had been using HTPs for a minimum of six months after quitting traditional cigarettes. The questionnaire was preceded by screening questions and by the explanation of the definition of HTPs, so that respondents had no doubts about which product category the survey is asking about. The study excluded those who tried but did not continue HTPs or reported no benefits. Without a control group, causal conclusions are limited, and findings may not apply beyond Poland.

The survey questionnaire comprised a description of participants (age, gender, education level, place of residence and income) and several thematic sections addressing key aspects of respondents’ well-being, including physical condition, mental health, psychophysical condition, social functioning, perceived social pressure, addiction and its perceived harmfulness, overcoming addiction, and environmental issues. Each thematic section followed a standardised structure consisting of three parallel question types: (1) assessment of the impact of smoking traditional cigarettes on the given aspect, (2) assessment of the impact of using HTPs on the same aspect, and (3) direct comparison of the perceived effects of both product types. In the present paper we have analysed questions related to physical health (Q10–Q15), which employed five-point categorical scales (ranging from *“Very good”* to *“Very poor”* or *“Had no impact”*) and comparative options such as *“My condition was better when I smoked traditional cigarettes”* versus *“My condition is better when I use heated tobacco products.”* Additionally, respondents were asked to indicate specific physical symptoms they experienced in association with traditional cigarette smoking and heated tobacco use (e.g., shortness of breath, cough, elevated blood pressure, heart palpitations, dizziness, nausea, nervousness, headaches, conjunctivitis, bad taste in the mouth, and discoloration of teeth or nails). These symptom-related questions allowed for a more detailed characterization of self-reported physical health effects associated with both product types. The detailed questionnaire is provided in the supplementary material.

The study population was examined across the following strata (predictor variables): gender, age, education, place of residence, and income. The source data on studies on age, education, residence, and income, originally divided into multiple categories (see Supplementary Material), were simplified for the purpose of this analysis into nominal variables, divided into two groups each: age groups: 18–39 years and 40 years and older; education groups: non-higher education (including incomplete primary, primary, lower secondary, basic vocational, incomplete secondary, secondary vocational, general secondary, post-secondary) and higher education (including incomplete higher, bachelor’s, master’s, and doctorate); place of residence groups: those living in rural areas or cities with up to 99,000 inhabitants, and those living in cities with more than 100,000 inhabitants; and income groups: below 5000 PLN per month and 5000 PLN or more per month.

This publication is focused on the segment of the survey related to physical condition.

### Statistical analysis

To estimate the frequency and relationships among categorical variables, descriptive statistics were employed. The Chi-square test of independence was used to assess the associations between independent variables (such as self-assessment of health, duration of smoking or HTP use, age at initiation of smoking or HTP use, age of initiation of daily smoking, attempts to quit smoking, identification of significant individuals in the respondents’ lives who smoked or used HTPs, and the impact of smoking or using HTPs on physical endurance and overall physical health) and various predictors (gender, age, education, place of residence, and income). Results were presented as frequencies and percentages, with statistical significance determined at *p* < 0.05. *Cramér’s V* coefficient was calculated to assess the strength of association between the impact of using HTPs and the impact of smoking traditional cigarettes on physical health. The impact of individual factors on respondents’ physical health was further analysed using odds ratios (ORs) and 95% confidence intervals (CIs), with differences deemed statistically significant at *p* < 0.05. To assess the combined effects of the predictors (sex, age, education, place of residence, and income) on respondents’ evaluation of the impact of smoking traditional cigarettes or using HTPs on their physical condition and overall physical health, a multinomial logistic regression model was constructed. Statistical analyses were conducted using JAMOVI software, version 2.4.14 and PQStat v.1.8.6 .

## Results

### Research group specification

The study included 2500 participants (1565 women and 935 men, representing 62.6% and 37.4% of the study population, respectively). The mean age of the respondents was 37 years, ranging from 18 to 83 years. The 18-39-year age group included 1559 people (62.36%) and the group 40 years and older 941 people (37.64%). Respondents with higher education made up 52.32% of the population (1308 people), while the remaining 47.68% (1192 people) had non-higher education. The population living in rural areas or cities with up to 99,000 inhabitants comprised 46.96% of the study population (1174 people), and those living in cities with more than 100,000 inhabitants, made up 53.04% of the study population (1326 people). The income group below 5000 PLN per month included 74.16% of the population (1854 people), while the group 5000 PLN or more per month accounted for 25.84% (646 people). The detailed description of the surveyed population is given in the Table S1.

In the studied cohort, the mean duration of traditional cigarette smoking was 11.5 years, while the use of HTPs averaged 2.5 years. Participants typically began smoking traditional cigarettes at the mean age of 21 and transitioned to HTPs around the age of 34. Most respondents transitioned to daily smoking within two years of initiation and, on average, made three attempts to quit. The respondents were also asked to identify individuals significant in their lives who smoked traditional cigarettes or used HTPs. For traditional cigarettes, 60.7% indicated their friends, while 54.3% mentioned their parents. Although the figures for HTPs were lower, a notable 51.8% of participants still cited their friends as people who use these products.

### Reported general health status

**Fig. 1:**
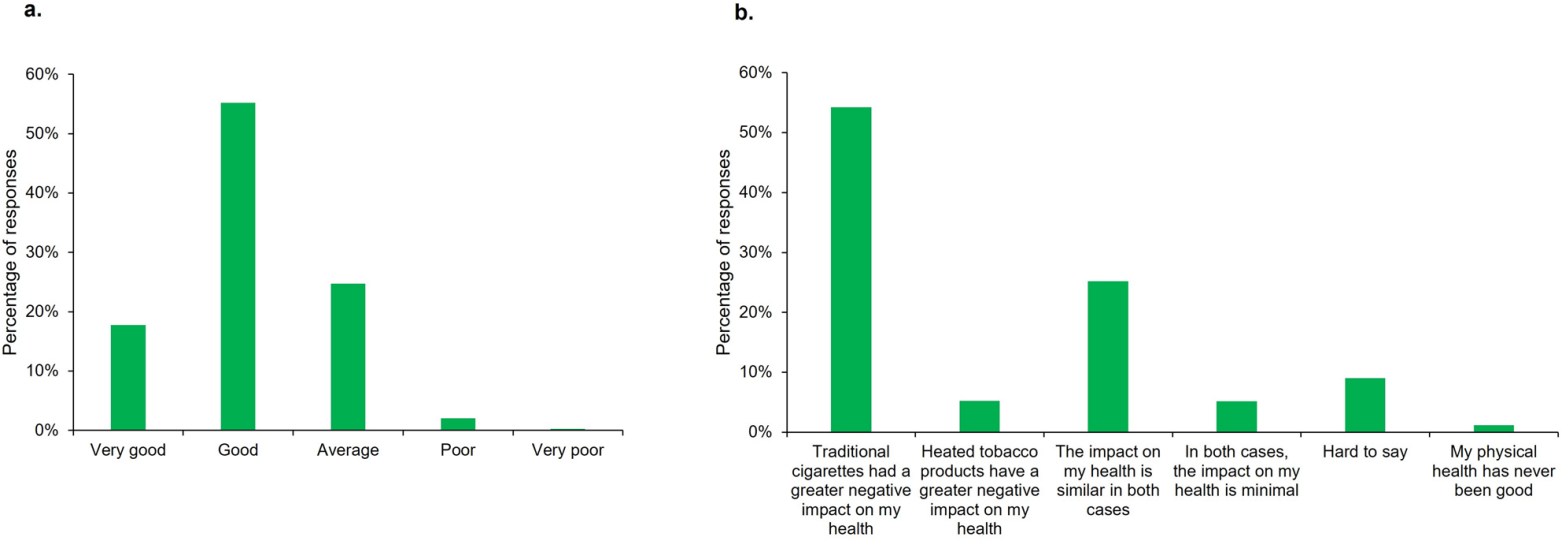
Reported general health status (**a**) and their opinion on the effects of traditional cigarettes and heated tobacco products on physical health (**b**)

Most respondents, despite smoking, rated their health as “Good” and “Very good” (The detailed percentage distribution of responses is presented in Fig. 1a). However, individuals who self-assessed their health as “Average,” “Poor,” or “Very poor” had a longer average duration of smoking traditional cigarettes (13.98 years; SD, 10; *p* < 0.05) compared to those who rated their health as “Good” or “Very good” (10.56 years; SD, 8; *p* < 0.05). A similar pattern emerged among individuals using HTPs, with poorer health correlating with longer usage (2.42 years; SD, 2.01; *p* < 0.05 vs. 2.39 years; SD, 1.84; *p* < 0.05) and more frequent quit attempts (2.8; SD, 1.13; *p* < 0.05 vs. 2.59; SD, 1.17; *p* < 0.05).

Both genders predominantly selected “Good” for their health status, however, a higher proportion of women reported “Very good” health (20.1%) compared to men (13.8%), while men were more likely to rate their health as “Average” (30.1% vs. 21.2%). Age-adjusted analysis revealed that younger participants (18–39 years) were, not surprisingly, more likely to report “Very good” health (23%) than those aged 40 and older (9%). Similarly, educational attainment influenced health perceptions, with those holding higher education degrees more frequently describing their health as “Very good” (19.3%) or “Good” (55.3%) compared to those with lower educational levels (16% and 55.1%, respectively). Income level also played a significant role; individuals earning above 5000 PLN per month were more likely to rate their health as “Very good” (21.4%) or “Good” (55.6%) compared to those with lower incomes (16.5% and 55.1%, respectively). Additionally, participants with lower incomes were more inclined to rate their health as “Poor” (2.3% vs. 1.4%). All the differences mentioned above were statistically significant (*p* < 0.05). The comparison of patients reported general health status between investigated groups is presented in Fig. S2 in supplementary materials.

### The comparison of the effects of traditional cigarettes and heated tobacco products on physical health

Most respondents highlight traditional cigarettes as being worse than HTPs for their physical health (the detailed percentage distribution of responses is presented in Fig. 1b). Older respondents (aged 40 and above) were more inclined to believe that than younger (61.1% vs. 50.1%, *p* < 0.05). Younger participants more frequently than older viewed HTPs as more harmful (6.1% vs. 3.8%, *p* < 0.05) or rated the impact of both similarly (27.6% vs. 21.1%, *p* < 0.05). The detailed comparison of respondents’ opinion of the effects of traditional cigarettes and HTPs on physical health in all investigated groups is presented in Fig. S3 in supplementary materials.

In line with the descriptive findings, the logistic regression analysis confirmed the effect of age on respondents’ evaluations of HTP-related harm. After adjusting for sex, older participants (≥ 40 years) were 42.7% less likely than younger individuals to report that HTPs had a greater negative impact on their health (Exp(B) = 0.573). The model also identified an independent association with sex: men were 47.4% less likely than women to express this view (Exp(B) = 0.526). These results indicate that both age and sex independently influence perceptions of the relative harm of HTPs in the studied population. The full numerical results of the multinomial logistic regression model are presented in Table S2 in the supplementary material.

### Reported effects of traditional cigarettes on physical condition

**Fig. 2:**
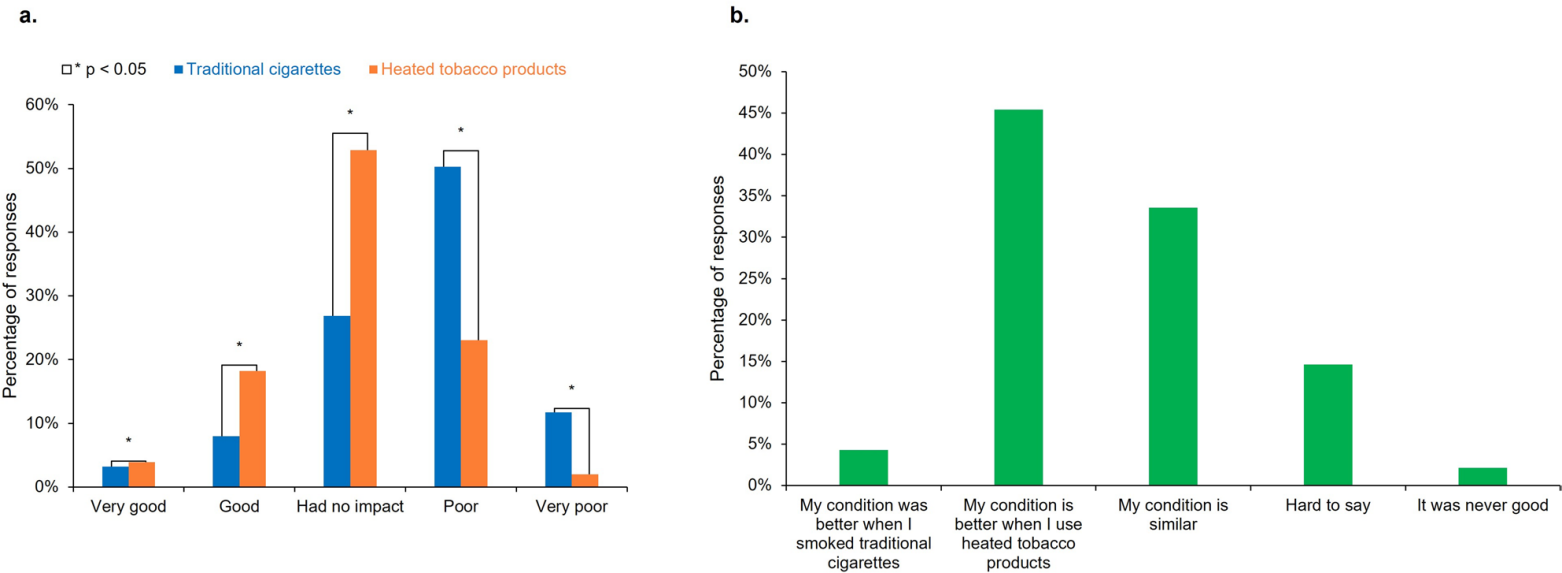
Reported effects of traditional cigarettes and heated tobacco products on physical condition (**a**) and comparison of both (**b**). *Abbreviations*: p - *P* value.

Most respondents (50.2%, presented in Fig. 2a) perceived impact of traditional cigarettes on their physical condition as poor. Gender-adjusted analysis showed that both men and women predominantly reported this poor effect, however women were more than man inclined to report “No impact” (28.7% vs. 23.7%; *p* < 0.05). Younger respondents (aged 18–39) more likely to report “No impact” on physical health (29.1% vs. 23.1%, *p* < 0.05), while older individuals (aged 40 and above) overwhelmingly indicated a “Poor” impact (54.4% vs. 47.7%, *p* < 0.05). The detailed comparison of self-reported effects of traditional cigarettes on physical condition between investigated groups is presented in Fig. S4 in supplementary materials.

### Reported effects of heated tobacco products on physical condition

Most respondents (52.9%) reported that HTPs did not affect their condition (the detailed percentage distribution of responses is presented in Fig. 2a). Statistically significant differences emerged across gender, age, and education. Women were more likely to report “No impact” (55.3% vs. 48.8%, *p* < 0.05), whereas men were more likely to perceive a positive effect on stamina (20.2% vs. 17%, *p* < 0.05). Interestingly, older respondents were more inclined to report a positive impact on condition compared to younger individuals (20.5% vs. 16.8%, *p* < 0.05). Additionally, those with higher education levels were more likely to emphasize the adverse effects of HTPs (25.6% vs. 20.2%, *p* < 0.05). The comparison of self-reported effects of HTPs on physical condition between investigated groups is presented in Fig. S5 in supplementary materials.

### The comparison of the effects of traditional cigarettes and heated tobacco products on physical condition

When comparing the effects of traditional cigarettes and HTPs on physical condition, a significant portion of respondents (the detailed percentage distribution of responses is presented in Fig. 2b) indicated that HTPs had a more favourable impact. Younger respondents (18–39 years) were more inclined than older ones to perceive both products as having a similar impact on their physical condition. (36.4% vs. 28.9%, *p* < 0.05), whereas older individuals (40 years and above) tended to report an improvement when using HTPs (51% vs. 42%, *p* < 0.05). Interestingly, participants with higher incomes, compared to those with lower incomes, more often reported an improvement in their condition with traditional cigarettes (6.7% vs. 3.5%, *p* < 0.05). The detailed comparison of respondents’ opinion on the effects of traditional cigarettes and HTPs on their physical condition in investigated groups is presented in Fig. S6 in supplementary materials.

The multinomial logistic regression analysis showed that men, older respondents and those with higher income differed significantly in how they evaluated their physical condition in relation to tobacco use (reference category: “Hard to say”). Respondents who indicated that *their condition was better when smoking traditional cigarettes* were less likely to be men (men were 55.1% less likely than women; Exp(B) = 0.449; *p* = 0.00064) and less likely to be older adults (older respondents were 40.8% less likely than younger individuals; Exp(B) = 0.592; *p* = 0.03029), whereas individuals with higher income were more likely to hold this view (88% more likely; Exp(B) = 1.880; *p* = 0.01188). Among those who reported that *their condition is better when using heated tobacco products*, men were again less likely to choose this option (40.6% less likely; Exp(B) = 0.594; *p* = 0.00011), while respondents with higher education were more likely to do so (28.7% more likely; Exp(B) = 1.287; *p* = 0.04591). For the response *“my condition is similar*,*”* men (37.4% less likely; Exp(B) = 0.626; *p* = 0.00089) and older individuals (27.7% less likely; Exp(B) = 0.723; *p* = 0.01473) were again less inclined to select this option, whereas participants with higher education were more likely to report similar condition regardless of product used (32.0% more likely; Exp(B) = 1.320; *p* = 0.03489). (Detailed results of the multinomial logistic regression analysis can be found in Table S3 in the supplementary material). Although these findings indicate several statistically significant associations, they are challenging to interpret because each outcome category is compared exclusively with the reference option rather than with each other. This structure yields *relative* effects that may differ across comparisons, producing patterns that can appear inconsistent. The low explanatory value of the model (McFadden’s R² = 0.0119) further suggests that demographic factors account for only a small portion of the variation in perceived physical condition. Thus, although some associations are statistically significant, they are weak, and the descriptive differences between groups may appear different from the regression results.

### The impact of traditional cigarettes and heated tobacco products on physical health symptoms

**Fig. 3:**
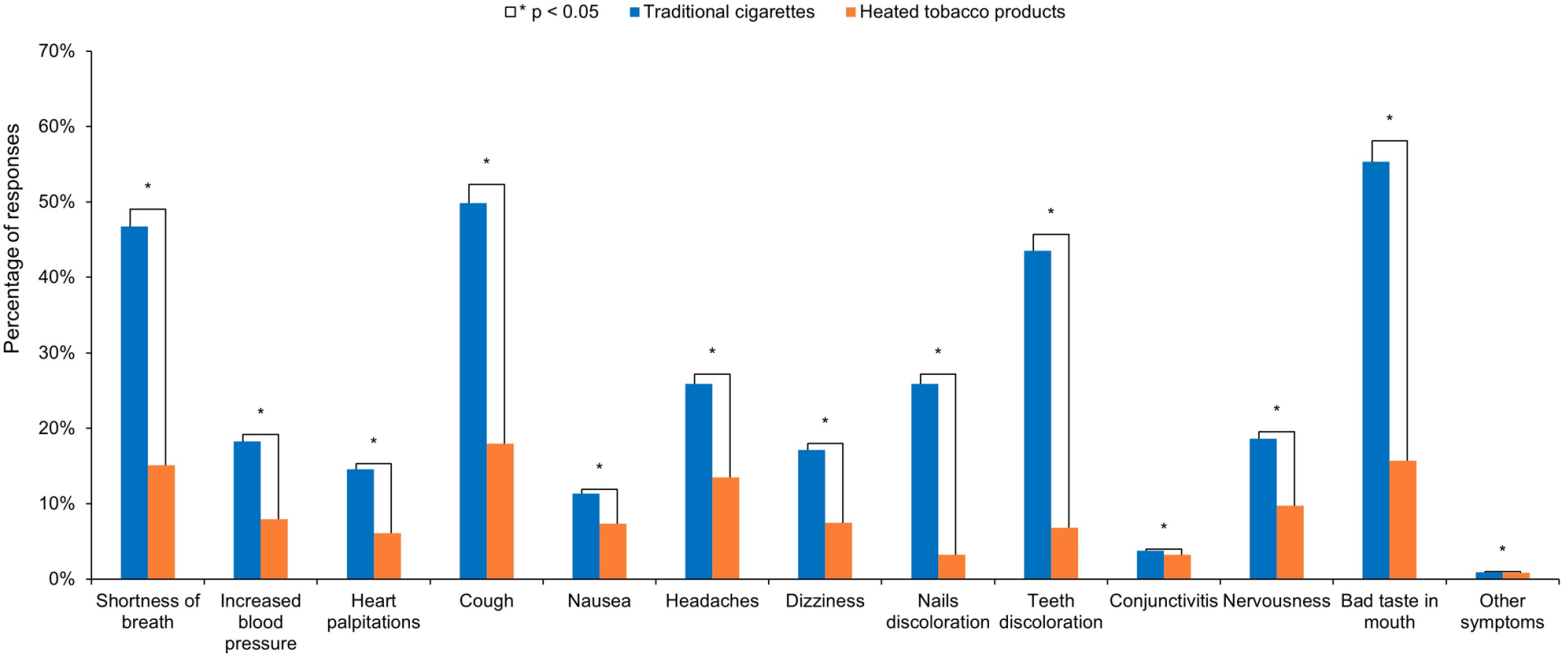
The impact of traditional cigarettes and heated tobacco products on physical health symptoms

In the perception of individuals who smoke traditional cigarettes have a significantly more pronounced negative impact on a broad range of physical health symptoms, as compared to HTPs. Only 10% responders stated that smoking traditional cigarettes did not cause any negative sensations for them. In contrast, 43% of respondents declared that they did not feel any negative impact on their physical health due to the use of HTPs. Respondents provided detailed feedback on the occurrence of specific symptoms, with the most frequently reported being a bad taste in the mouth (71% overall, 55.3% for traditional cigarettes) and coughing (67.8% overall, 49.8% for traditional cigarettes). In comparison to HTPs smoking traditional cigarettes was also more often related to shortness of breath, nervousness, yellowing of teeth and nails. The frequency of various physical symptoms in study population is presented on Fig. 3.

**Fig. 4:**
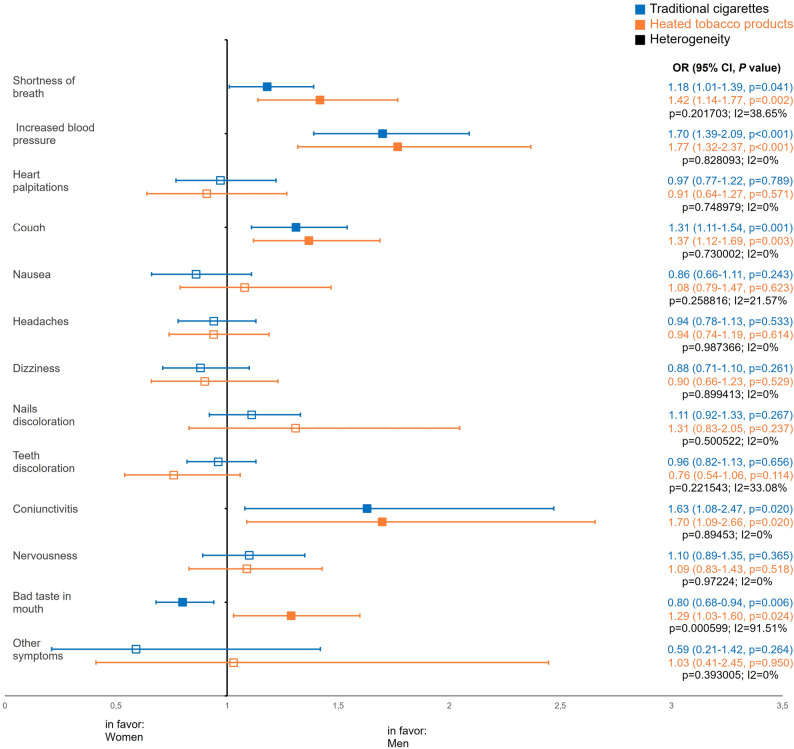
Comparison of the impact of traditional cigarettes and heated tobacco products on reported physical health symptoms. Adjustment for gender. *Abbreviations*: OR, odds ratio: CI, confidence interval; p, *P* value

A gender-adjusted analysis (Fig. 4) demonstrated that women were more prone to experience a bad taste in the mouth when smoking traditional cigarettes (OR 0.8, *p* < 0.05), while men more commonly reported this symptom with HTPs (OR 1.29, *p* < 0.05). Additionally, men had a significantly higher likelihood of experiencing symptoms such as shortness of breath (OR 1.18, *p* < 0.05), elevated blood pressure (OR 1.7, *p* < 0.05), cough (OR 1.31, *p* < 0.05), and conjunctivitis (OR 1.63, *p* < 0.05) when smoking traditional cigarettes, as well as when using HTPs (OR 1.42, *p* < 0.05; OR 1.77, *p* < 0.05; OR 1.37, *p* < 0.05; OR 1.7, *p* < 0.05, respectively).

**Fig. 5:**
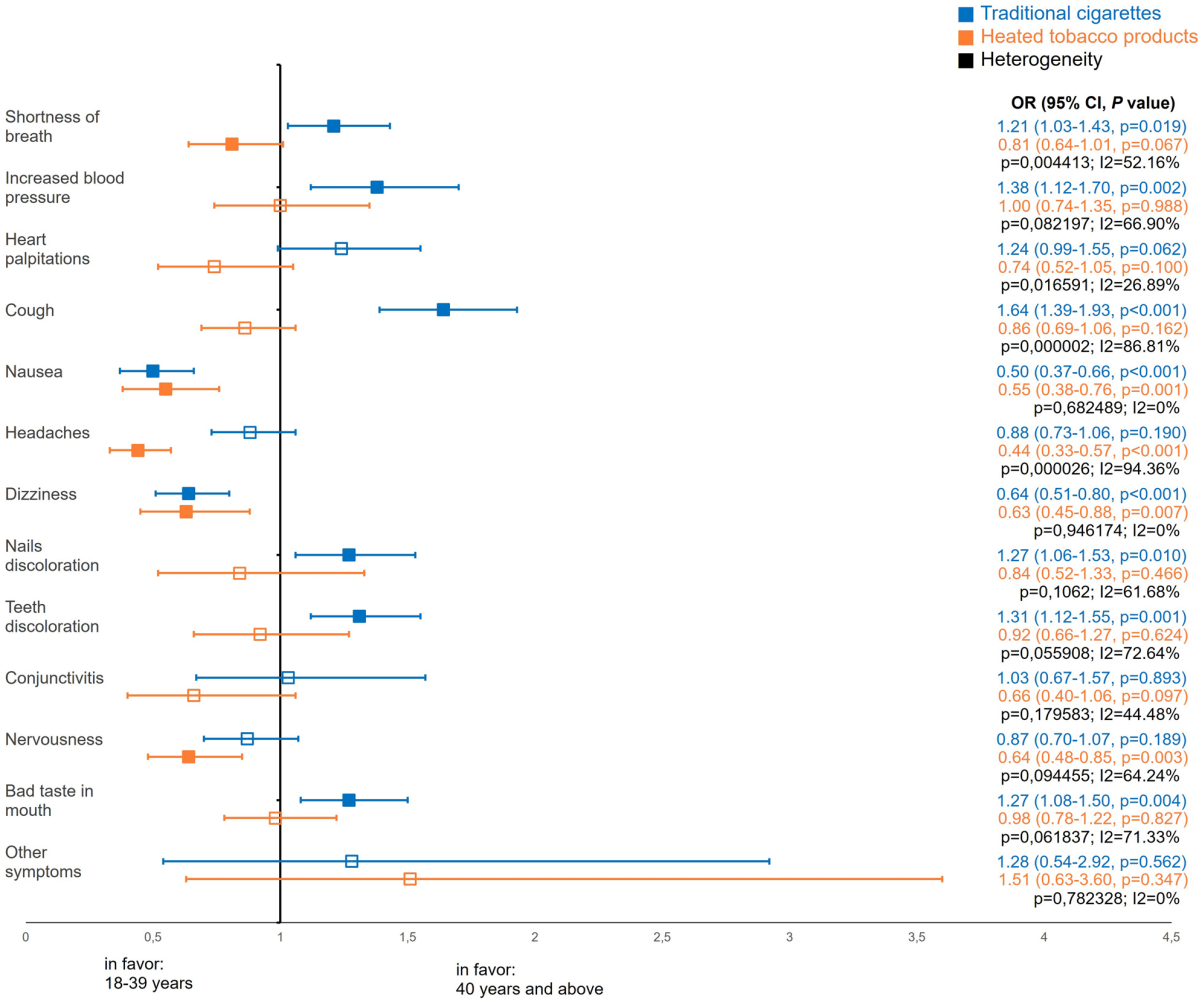
Comparison of the impact of traditional cigarettes and heated tobacco products on reported physical health symptoms. Adjustment for age. *Abbreviations*: OR, odds ratio: CI, confidence interval; p, *P* value

Age-related trends (Fig. 5) revealed that older respondents were more prone to experiencing shortness of breath (OR 1.21, *p* < 0.05), increased blood pressure (OR 1.38, *p* < 0.05), coughing (OR 1.64, *p* < 0.05), nail discoloration (OR 1.27, *p* < 0.05), teeth discoloration (OR 1.31, *p* < 0.05), and an unpleasant taste in the mouth (OR 1.27, *p* < 0.05) when smoking traditional cigarettes, compared to younger individuals. Conversely, younger respondents were more likely to report nausea (OR 0.5, *p* < 0.05) and dizziness (OR 0.64, *p* < 0.05) when smoking traditional cigarettes, as well as when using HTPs (OR 0.55, *p* < 0.05; OR 0.63, *p* < 0.05, respectively). Additionally, younger individuals more frequently experienced nervousness (OR 0.64, *p* < 0.05) and headaches (OR 0.44, *p* < 0.05) in association with HTPs.

**Fig. 6:**
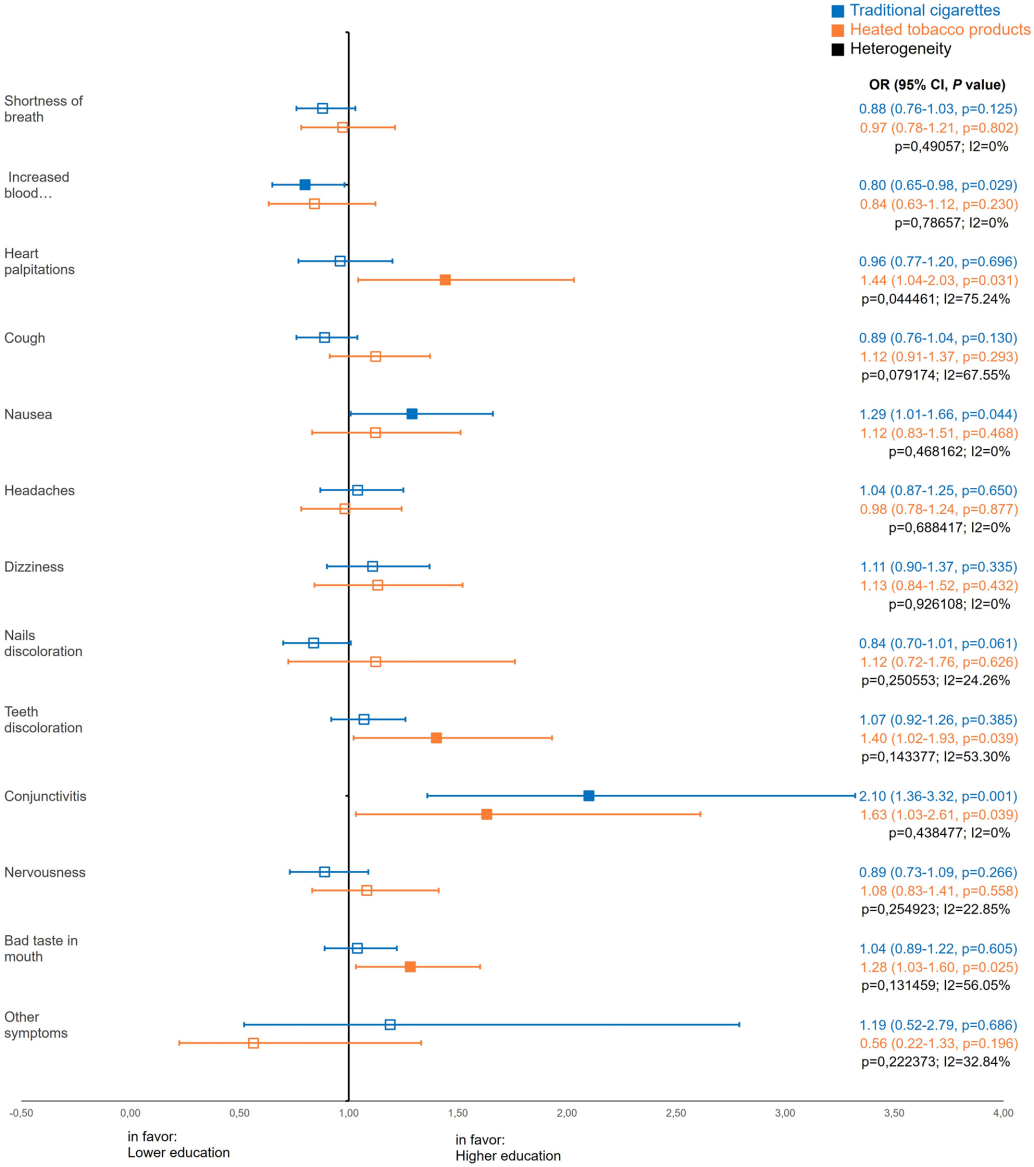
Comparison of the impact of traditional cigarettes and heated tobacco products on reported physical health symptoms. Adjustment for education. *Abbreviations*: OR, odds ratio: CI, confidence interval; p, *P* value

Differences in education level further influenced symptom reporting (Fig. 6). Individuals with higher education were more likely to report conjunctivitis (OR 2.1, *p* < 0.05) and nausea (OR 1.29, *p* < 0.05) when smoking traditional cigarettes, while those with lower education were more prone to reporting increased blood pressure (OR 0.8, *p* < 0.05). In the case of HTPs, higher-educated individuals more frequently observed heart palpitations (OR 1.44, *p* < 0.05), teeth discoloration (OR 1.4, *p* < 0.05), conjunctivitis (OR 1.63, *p* < 0.05), and an unpleasant taste in the mouth (OR 1.28, *p* < 0.05) compared to their lower-educated counterparts. Conversely, lower-educated individuals reported increased blood pressure more often (OR 0.84, *p* < 0.05).

**Fig. 7:**
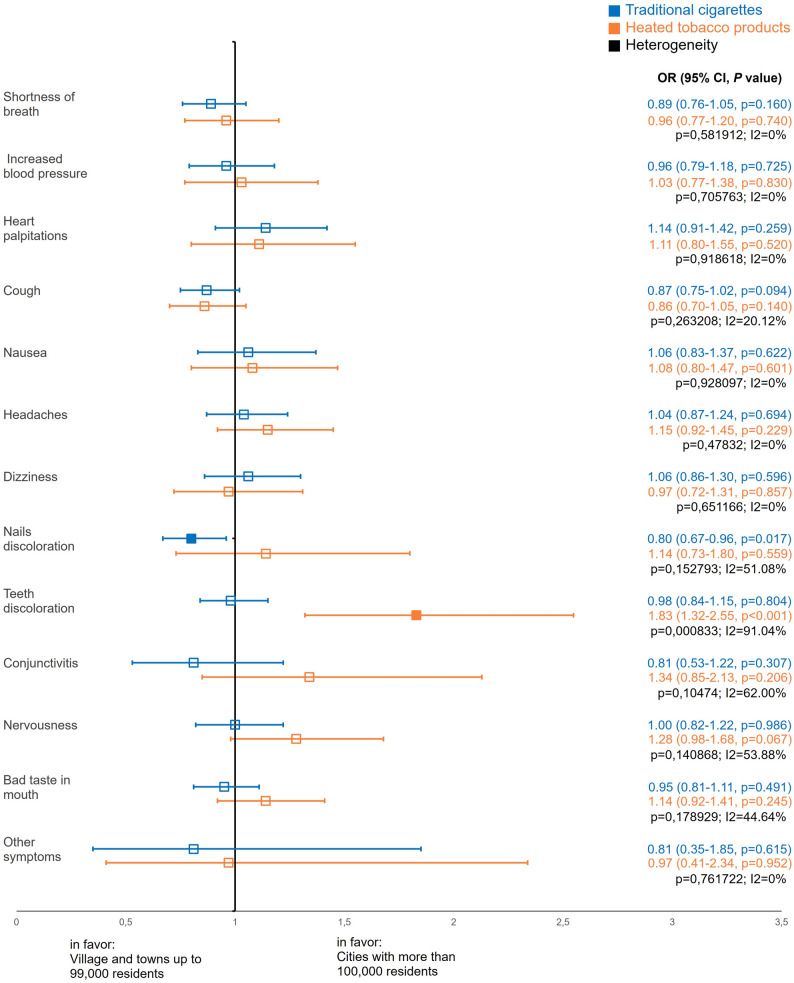
Comparison of the impact of traditional cigarettes and heated tobacco products on reported physical health symptoms. Adjustment for place of residence. *Abbreviations*: OR, odds ratio: CI, confidence interval; p, *P* value

Place of residence also played a role (Fig. 7). Individuals living in rural areas or cities with populations up to 99,000 were more likely to experience nail discoloration from traditional cigarette use (OR 0.8, *p* < 0.05), while those residing in larger cities more frequently reported teeth discoloration associated with HTPs (OR 1.83, *p* < 0.05).

**Fig. 8:**
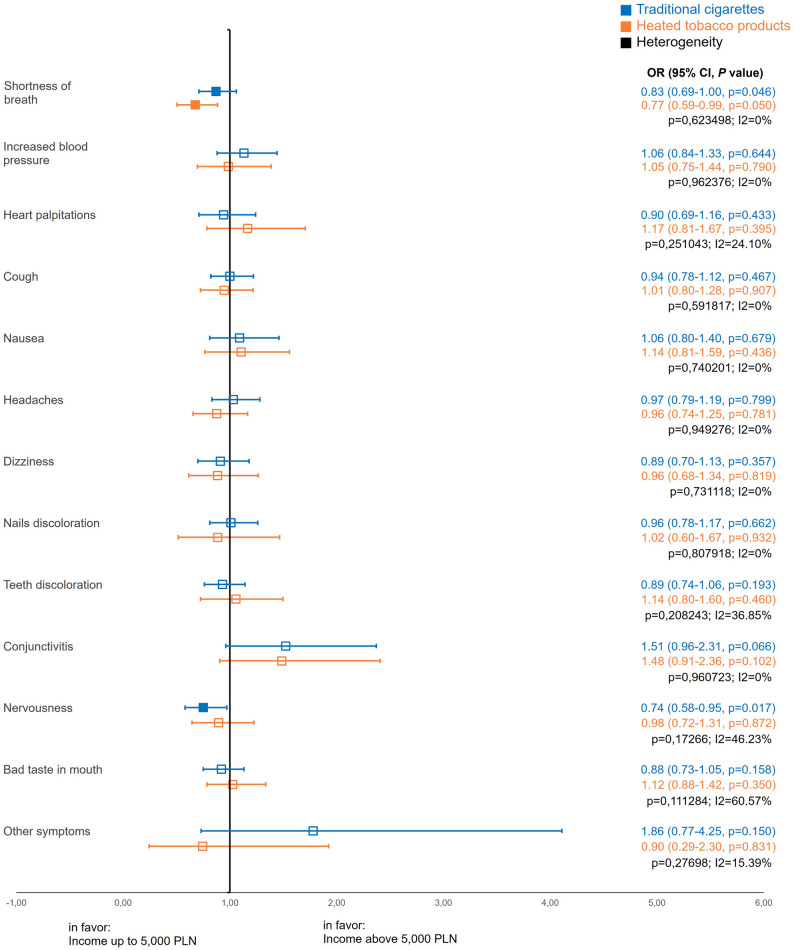
Comparison of the impact of traditional cigarettes and heated tobacco products on reported physical health symptoms. Adjustment for income. *Abbreviations*: OR, odds ratio: CI, confidence interval; p, *P* value

Finally, income levels influenced health perceptions and symptoms (Fig. 8), with lower-income individuals showing a significantly higher likelihood of reporting shortness of breath with both traditional cigarettes (OR 0.83 *p* < 0.05) and HTPs (OR 0.77, *p* < 0.05). Additionally, they were more likely to report nervousness associated with traditional cigarette use (OR 0.74, *p* < 0.05).

## Discussion

This study showed that most adults who currently use HTPs, having switched from traditional cigarettes, perceive their overall health as good. Respondents highlighted traditional cigarettes as being worse than HTPs for their general health status (Fig. 1). With regard to physical condition, the majority of study participants thought that HTPs do not affect this aspect of health, whereas impact of traditional cigarettes on physical endurance was rated mostly as negative or very negative (Fig. 2). Study participants also indicated that traditional cigarettes have a more pronounced impact on a broad range of physical health symptoms (such as bad taste in the mouth, cough, shortness of breath, high blood pressure, heart palpitations, headaches, dizziness, nervousness, and yellowing of teeth and nails) compared to HTPs (Fig. 3). It is worth noting that the study included only individuals who had switched from smoking to exclusive HTP use for at least six months, excluding those who discontinued. Without a control group and given the Polish-only sample, the findings are not broadly generalizable.

Various studies have shown that the aerosol from HTPs may be less toxic than the smoke from traditional cigarettes. These findings are based on experiments involving human bronchial epithelial cells, coronary arterial endothelial cells, gingival epithelial cultures, monocytic cells, and mouse models [14]. Most studies also show that switching from conventional cigarettes to HTPs leads to notable decreases in levels of biomarkers linked to the cardiovascular disease pathways, such as inflammation, oxidative stress, lipid metabolism, platelet function, and endothelial dysfunction. This reduction might indicate a lower risk of developing diseases associated with smoking [15, 16]. Therefore, it appears that the well-being of individuals who switch from traditional cigarettes to HTPs may improve, their physical endurance may increase, and adverse physical health symptoms may occur less frequently.

Indeed, a recent, cross-sectional, web-based Hungarian survey, showed that individuals who exclusively use HTPs may experience the beneficial health effects of switching from smoking to HTPs. More than half of people who exclusively use HTPs reported improvements in morning cough, breathing, smell, physical status in general, and tooth discoloration after the switch [17]. These results seem to be similar to those presented in our study. Furthermore, a cross-sectional, school-based survey conducted in Hong Kong among young people (33 627 students, mean age 14.8 years), who used HTPs, revealed that HTP use was associated with respiratory symptoms, especially in those who had never used cigarettes. In people who previously smoked traditional cigarettes, current HTPs use was still associated with higher risks of symptoms compared to those who had never used HTPs. However, this study did not evaluate the intensity of symptoms after switching from traditional cigarettes to HTPs [18]. The use of HTPs, compared with individuals who do not use nicotine products, may be associated with an increased incidence of respiratory disorders, including impaired lung function, altered bronchial epithelial cell activity, and conditions such as acute eosinophilic pneumonia, allergic rhinitis, and asthma [16]. It may also contribute to increased oxidative stress [19], mitochondrial dysfunction [20], and a higher frequency of respiratory tract infections [21] compared with individuals who do not use nicotine. However, traditional cigarettes exert an even greater detrimental impact on the respiratory system and are more strongly associated with the development and progression of respiratory diseases than HTPs. Consistently, many respondents in our study continued to report respiratory symptoms such as shortness of breath and coughing associated with HTP use (Fig. 3); however, the prevalence of these symptoms was markedly lower than that observed with traditional cigarette smoking.

It cannot be ruled out that our respondents’ perception of traditional cigarettes as being more harmful than HTPs may be influenced by their personal beliefs. Limited data exist on the public awareness and perceptions regarding HTPs. The study including Hungarian adults with a history of HTP use, either past or current showed that as many as 86.2% of the participants perceived HTPs to be less harmful that traditional cigarettes. They demonstrated distorted risk perceptions concerning both HTPs and e-cigarettes [22]. On the other hand, in Korean survey among adults who smoke cigarettes, it was shown that only 27.5% of them believe that HTPs are less harmful than cigarettes [23]. It is of course possible that the perceived harmfulness of HTPs differs across countries.

The perception of harmfulness may be related also to the smoking status and some demographic factors. A Japanese study showed that 40.3% of individuals who currently use tobacco perceived HTPs as less harmful, compared to 18.3% among those who do not use tobacco. Among individuals who do not use tobacco, the male gender, age under 39 years, and lower education were associated with the perception of HTPs as less harmful [24]. In turn, in a Hungarian study, among people who currently use HTPs older age was associated with significantly greater odds of perceiving HTPs as less harmful than traditional cigarettes [22]. Likewise in our study older respondents were more than younger ones inclined to believe that traditional cigarettes are worse than HTPs for their physical health and tended to report an improvement in their physical condition when using HTPs. Compared to younger individuals, older respondents were more prone to report shortness of breath, increased blood pressure, coughing, nail discoloration, teeth discoloration, and an unpleasant taste in the mouth when smoking traditional cigarettes. In addition to age, other demographic factors such as sex and income also showed some associations with perceptions of HTP-related harm, although their effects were modest. Overall, these findings suggest that while demographic variables play a role, individual perceptions are likely shaped by a broader range of personal experiences, health beliefs, and lifestyle factors. It should be noted that the patterns observed in regression analyses may not exactly mirror the descriptive percentages, since each category is compared only to the reference option. This highlights that individual perceptions are influenced by multiple factors beyond the demographic variables captured in this study.

We can only speculate on the reasons for the observed age-related differences in the perception of smoking’s harmfulness and physical health symptoms. They may be attributed to the multi-morbidity often observed in older individuals or those with generally worse health, which makes the frequency of their signs and symptoms higher, and differences more perceivable. Additionally, older adults tend to have a greater interest in health-related matters and frequently seek health information from various sources, including the internet. Tobacco companies often use marketing that emphasizes the reduced harm of HTPs [25]. This type of marketing can influence perceptions, particularly among older adults who may be looking for ways to reduce harm while continuing to use tobacco. Older individuals might also be less familiar with the technology behind HTPs and therefore more likely to believe claims that these products are less harmful without fully understanding the science.

Our study has some limitations. Like all survey research, it may be biased by various factors. Some of these are:

- Cognitive misinterpretation: respondents may experience difficulties in comprehending the survey questions, which can result in inaccurate or incomplete data collection,
- Response bias: a lack of intrinsic motivation among respondents may lead to the provision of socially desirable answers rather than truthful responses, thereby compromising data integrity, additionally individuals with more positive experiences and perceptions towards HTPs may have been more motivated to participate in the study,
- Social desirability bias: respondents might feel discomfort in disclosing information that could reflect negatively on them, leading to biased or skewed data,
- Ambiguity in response options: the variability in the interpretation of response options by different respondents can introduce inconsistencies and ambiguities in the collected data,
- Subjectivity in responses: the inherent subjectivity in respondents’ answers can affect the objectivity and reliability of the survey results.

Additionally, all respondents were individuals who formerly smoked traditional cigarettes and had successfully transitioned to using HTPs and maintained this change for at least six months. This highly selected sample excludes individuals who may have tried HTPs but did not continue using them or did not perceive health benefits, and importantly, the study lacks a control group of such individuals. This limits the generalizability of our findings and precludes causal inferences regarding health outcomes. Some reported symptoms may have persisted from prior cigarette smoking but were attributed to HTP use.

Another limitation is that no detailed information was collected regarding the specific types or brands of HTPs used by respondents. Since different HTP devices may vary in heating mechanisms, temperature profiles, and emissions, this factor could influence the level of toxicant exposure and, consequently, perceived health effects.

Finally, the study population is restricted to Poland, and thus the results may not be generalizable to other countries or cultural contexts.

However, this study is one of the very few that aim to assess physical symptoms that may be related to the use of HTPs. The quality and reliability of the answers are confirmed by the fact that the survey was performed by two independent institutions, and the results were similar. As randomized clinical studies directly comparing traditional cigarettes and HTPs are ethically challenging, and long-term epidemiological data are still lacking, surveys may serve as a valuable complementary source of information.

We conclude that most individuals who currently use HTPs and have switched from traditional cigarettes perceive traditional cigarettes as more harmful to their physical health than HTPs. Most also believe that traditional cigarettes more negatively affect physical endurance and various physical signs and symptoms. Age appears to be the main demographic factor influencing these opinions, with older respondents being less likely than younger individuals to report greater harm from HTPs. While other factors such as sex, income, and education showed some associations in regression analyses, their effects were small, indicating that demographic variables explain only a minor part of the variation in perceptions.

## Supplementary Information

Below is the link to the electronic supplementary material.

Supplementary Material 1

## Data Availability

The data that support the findings of this study are available from the corresponding author, [MW], upon reasonable request.
