# Supplementary material for: Self-reported physical health among people who switched from traditional cigarettes to heated tobacco products: a large-scale national survey
Source: Harm Reduct J. 2026 May 6;23:121. doi: 10.1186/s12954-026-01466-2 (PMC13374294; doi:10.1186/s12954-026-01466-2)
Supplement: Supplementary file 1 — Supplementary Material 1 [file 12954_2026_1466_MOESM1_ESM.docx]

**Supplementary material**

**Material and Methods**

**The research plan was executed in close collaboration with experts from the Public Opinion Research Center (CBOS) in Poland, who were integral in designing the primary research instrument - the survey questionnaire - and developing its electronic version for respondent use. Upon completion of this preparation, CBOS delegated the tool for direct implementation of the research, utilizing the CAWI (Computer Assisted Web Interview) method. The research was conducted by two independent institutes: IQS Think Forward sp. z o.o., a leader in online market research in Poland, and Pollster, a specialist in research using advanced technologies. These institutes were tasked with conducting the survey electronically, gathering the final data, and submitting it to CBOS as the lead partner.** The study involved two parallel surveys: one by IQS Think Forward sp. z o.o., surveying 1,250 adults using a questionnaire refined in collaboration with CBOS experts, and another by Pollster, surveying an additional 1,250 adults using the same questionnaire. To ensure the research's objectivity, both institutes were provided with identical questionnaires, utilizing the same scripts, iconography, and typographic layout. Independent recruitment was conducted by each institute from their respective respondent groups, ensuring that both groups were equal in size, with 1,250 respondents each. In both cases the potential participants were selected from the smokers – including databases of the given institute and invited to the survey.

The survey included 2,500 adult participants who had smoked traditional cigarettes for at least a year and had used heated tobacco products (HTBs) for a minimum of six months after quitting cigarettes. This methodology allowed respondents to evaluate and compare their physical and mental well-being during their smoking period with their current state while using HTBs. The specified duration - at least one year of smoking followed by six months of HTB use - provided respondents with sufficient time to form a well-considered opinion regarding the impact of these products on their overall well-being. This timeframe was also sufficient for participants to assess whether the previous product had a greater, lesser, or comparable effect on their well-being relative to the current product.

The survey comprised 67 questions divided into several key sections, including preliminary questions, physical condition, mental condition, psychophysical condition, social condition, social pressure, addiction and its perceived harmfulness, overcoming addiction, environmental concerns, and concluding questions. The survey was preceded by screening questions and a clear definition of HTPs to ensure that respondents understood the product category being discussed. To maximize research objectivity, comparative studies were employed; thus, each survey segment included three components: assessing the impact of smoking on the issue, evaluating the impact of using heaters, and comparing the effects of both products. This article focuses on analyzing the first group of 15 questions related to respondents’ reported physical health.

The study population was stratified by gender, age, education, place of residence, and income. The original data, categorized into multiple groups based on age, education, residence, and income, were simplified into nominal variables with two groups each. Age was divided into 18-39 years and 40 years and older. Education was categorized into non-higher education (**including incomplete primary, primary, lower secondary, basic vocational, incomplete secondary, secondary vocational, general secondary, post-secondary)** and higher education (**including incomplete higher, bachelor’s, master’s, and doctorate**). Residence was classified into rural areas or cities with up to 99,000 inhabitants, and cities with more than 100,000 inhabitants. Income was split into below 5,000 PLN per month and 5,000 PLN or more per month. The detailed characteristics of the study group are presented in Table S1.

The study included 1,565 women (62.6%) and 935 men (37.4%), with an average respondent age of 37 years, ranging from 18 to 83 years. The 18-39 age group comprised 62.36% of the sample (1,559 individuals), while the 40+ age group made up 37.64% (941 individuals). Participants with higher education accounted for 52.32% (1,308 individuals) of the sample, while those with non-higher education comprised 47.68% (1,192 individuals). The population living in rural areas or cities with up to 99,000 inhabitants represented 46.96% of the sample (1,174 individuals), and those residing in cities with more than 100,000 inhabitants accounted for 53.04% (1,326 individuals). The income group earning below 5,000 PLN per month included 74.16% of the respondents (1,854 individuals), while the higher income group (5,000 PLN or more) represented 25.84% (646 individuals). Detailed data are presented in Table S1.

Table S1. Characteristics of the study group by Gender, Age, Education, Place of Residence, and Income

| **Gender** | **N (%)** | **Women N (%)** |
| --- | --- | --- |
| Women | 1,564 (62.6%) |  |
| Men | 936 (37.4%) |  |
| **Age (years)** |  |  |
| 18-29 | 663 (26.5%) | 508 (32.5%) |
| 30-39 | 896 (35.8%) | 561 (35.9%) |
| 40-49 | 550 (22.0%) | 309 (19.8%) |
| 50-59 | 261 (10.4%) | 131 (8.4%) |
| 60+ | 130 (5.2%) | 55 (3.5%) |
| **Education level** |  |  |
| Lower | 160 (6.4%) | 77 (4.9%) |
| Secondary | 1,032 (41.3%) | 641 (41.0%) |
| Higher | 1,308 (52.3%) | 846 (54.1%) |
| **Place of residence** |  |  |
| Rural areas | 360 (14.4%) | 247 (15.8%) |
| Cities up to 100,000 inhabitants | 814 (32.6%) | 475 (30.4%) |
| Cities with 100,000-500,000 inhabitants | 774 (31.0%) | 491 (31.4%) |
| Cities with more than 500,000 inhabitants | 552 (22.1%) | 351 (22.4%) |
| **Income** |  |  |
| No income | 98 (3.9%) | 70 (4.5%) |
| 0-3,000 PLN | 549 (22.0%) | 425 (27.2%) |
| 3,000-5,000 PLN | 1,207 (48.3%) | 756 (48.3%) |
| 5,000-10,000 PLN | 516 (20.6%) | 256 (16.4%) |
| 10,000 PLN or more | 130 (5.2%) | 57 (3.6%) |

**Survey questions**

S01. Have you smoked traditional cigarettes (regular, conventional) habitually for a period of at least one year?

S02. Have you quit smoking traditional cigarettes and switched to using heated tobacco products?

S03. How long have you been using heated tobacco products?

Q01. How would you rate your health condition?

1. Very good
2. Good
3. Average
4. Poor
5. Very poor

Q02. How many years did you smoke traditional cigarettes? If you don't remember exactly, please provide an approximate number rounded to the nearest year.

Q03. How many years have you been using only heated tobacco products? If you don't remember exactly, please provide an approximate number rounded to the nearest year.

Q04. How old were you when you started smoking traditional cigarettes? If you don't remember exactly, please provide an approximate number rounded to the nearest year.

Q05. How old were you when you started using heated tobacco products? If you don't remember exactly, please provide an approximate number rounded to the nearest year.

Q06. How quickly after smoking your first traditional cigarette did you start smoking daily?

Q07. How many times have you tried to quit smoking traditional cigarettes?

Q08. Which of the important people in your life smoke or have smoked traditional cigarettes?

Q08_01 - No one smokes/smoked

Q08_02 - Parents/guardians

Q08_03 - Grandparents

Q08_04 - Siblings

Q08_05 - Neighbours

Q08_06 - Closest friend

Q08_07 - Acquaintances

Q08_08 - Your teacher

Q08_09 - Your idol/role model

Q09. Which of the important people in your life use or have used heated tobacco products? You can select more than one answer.

Q09_01 - No one uses/used

Q09_02 - Parents/guardians

Q09_03 - Grandparents

Q09_04 - Siblings

Q09_05 - Neighbours

Q09_06 - Closest friend

Q09_07 - Acquaintances

Q09_08 - Your teacher

Q09_09 - Your idol/role model

Q10. How did smoking traditional cigarettes affect your physical condition (endurance)?

1. Very good
2. Good
3. Had no impact
4. Poor
5. Very poor

Q11. How does using heated tobacco products affect your physical condition (endurance)?

1. Very good
2. Good
3. Has no impact
4. Poor
5. Very poor

Q12. Compare the impact of smoking traditional cigarettes and using heated tobacco products on your physical condition (endurance).

1. My condition was better when I smoked traditional cigarettes
2. My condition is better when I use heated tobacco products
3. My condition is similar
4. Hard to say
5. It was never good

Q13. How did smoking traditional cigarettes affect your physical health? You can select more than one answer.

Q13_01 - It did not affect my physical health

Q13_02 - Shortness of breath

Q13_03 - Increased blood pressure

Q13_04 - Heart palpitations

Q13_05 - Cough

Q13_06 - Nausea

Q13_07 - Headaches

Q13_08 - Dizziness

Q13_09 – Nails discoloration

Q13_10 – Teeth discoloration

Q13_11 - Conjunctivitis

Q13_12 - Nervousness

Q13_13 - Bad taste in the mouth

Q13_14 - Other symptoms

Q14. How does using heated tobacco products affect your physical health? You can select more than one answer.

Q14_01 - It did not affect my physical health

Q14_02 - Shortness of breath

Q14_03 - Increased blood pressure

Q14_04 - Heart palpitations

Q14_05 - Cough

Q14_06 - Nausea

Q14_07 - Headaches

Q14_08 - Dizziness

Q14_09 – Nails discoloration

Q14_10 – Teeth discoloration

Q14_11 - Conjunctivitis

Q14_12 - Nervousness

Q14_13 - Bad taste in the mouth

Q14_14 - Other symptoms

Q15. Compare the impact of smoking traditional cigarettes and using heated tobacco products on your physical health.

1. Traditional cigarettes had a greater negative impact on my health
2. Heated tobacco products have a greater negative impact on my health
3. The impact on my health is similar in both cases
4. In both cases the impact on my health is minimal
5. Hard to say
6. My physical health has never been good

Q16. How did smoking traditional cigarettes affect your mental condition?

1. Very good
2. Good
3. Had no impact
4. Poor
5. Very poor

Q17. How does using heated tobacco products affect your mental health?

1. Very good
2. Good
3. Has no impact
4. Poor
5. Very poor

Q18. Compare the impact of smoking traditional cigarettes and using heated tobacco products on your mental health.

1. The effect was greater when I smoked traditional cigarettes
2. The effect is greater when I use heated tobacco products
3. In both cases, the effect is similar
4. Hard to say
5. In both cases, I don't feel any effect
6. My mental condition has never been good

Q19. Did smoking traditional cigarettes cause you any discomfort? If so, what were the symptoms? You can select more than one answer.

Q19_01 - It did not cause discomfort

Q19_02 - Agitation

Q19_03 - Distraction

Q19_04 - Irritability

Q19_05 - Insomnia

Q19_06 - Depression

Q19_07 - Melancholy

Q19_08 - Other symptoms

Q20. Does using heated tobacco products cause you any discomfort? If so, what are the symptoms? You can select more than one answer.

Q20_01 - It does not cause discomfort

Q20_02 - Agitation

Q20_03 - Distraction

Q20_04 - Irritability

Q20_05 - Insomnia

Q20_06 - Depression

Q20_07 - Melancholy

Q20_08 - Other symptoms

Q21. Compare your sense of discomfort resulting from smoking traditional cigarettes and using heated tobacco products.

1. I felt better when I smoked traditional cigarettes
2. I feel better when I use heated tobacco products
3. I feel similarly
4. Hard to say
5. It was never good

Q22. Did you have any negative feelings during the period when you smoked traditional cigarettes? If so, what were they? You can select more than one answer.

Q22_01 - I had no negative feelings

Q22_02 - Lowered self-esteem

Q22_03 - Anxiety about health

Q22_04 - Decreased motivation

Q22_05 - Feeling of helplessness

Q22_06 - Feeling misunderstood by others

Q22_07 - Guilt

Q22_08 - General pessimism

Q22_09 - Lack of confidence

Q22_10 - Shame

Q22_11 - Anger

Q22_12 - Other feelings

Q23. Did you have any negative feelings while using heated tobacco products? You can select more than one answer.

Q23_01 - I have no negative feelings

Q23_02 - Lowered self-esteem

Q23_03 - Anxiety about health

Q23_04 - Decreased motivation

Q23_05 - Feeling of helplessness

Q23_06 - Feeling misunderstood by others

Q23_07 - Guilt

Q23_08 - General pessimism

Q23_09 - Lack of confidence

Q23_10 - Shame

Q23_11 - Anger

Q23_12 - Other feelings

Q24. Compare the intensity of your negative feelings resulting from smoking traditional cigarettes and using heated tobacco products.

1. They were stronger when I smoked traditional cigarettes
2. They are stronger when I use heated tobacco products
3. Hard to say
4. They are equally strong
5. They are equally negligible

Q25. What positive feelings did you have during the period when you smoked traditional cigarettes? You can select more than one answer.

Q25_01 - I had no positive feelings

Q25_02 - A sense of reward

Q25_03 - A sense of relaxation

Q25_04 - A sense of uniqueness

Q25_05 - Time just for myself

Q25_06 - Other feelings

Q26. What positive feelings do you have when using heated tobacco products? You can select more than one answer.

Q26_01 - I have no positive feelings

Q26_02 - A sense of reward

Q26_03 - A sense of relaxation

Q26_04 - A sense of uniqueness

Q26_05 - Time just for myself

Q26_06 - Other feelings

Q27. Compare the intensity of your positive feelings resulting from smoking traditional cigarettes and using heated tobacco products.

1. They were stronger when I smoked traditional cigarettes
2. They are stronger when I use heated tobacco products
3. Hard to say
4. They are equally strong
5. They are equally negligible

Q28. How did you feel when you couldn't smoke a traditional cigarette? You can select more than one answer.

Q28_01 - It didn’t bother me

Q28_02 - Irritability

Q28_03 - Discouragement

Q28_04 - Accumulation of negative emotions

Q28_05 - Racing thoughts

Q28_06 - Chaotic thoughts

Q28_07 - Thinking about smoking a cigarette as soon as possible

Q28_08 - Other feelings

Q29. How do you feel when you can't use a heated tobacco product? You can select more than one answer.

Q29_01 - It doesn’t bother me

Q29_02 - Irritability

Q29_03 - Discouragement

Q29_04 - Accumulation of negative emotions

Q29_05 - Racing thoughts

Q29_06 - Chaotic thoughts

Q29_07 - Thinking about smoking a cigarette as soon as possible

Q29_08 - Other feelings

Q30. Please compare the intensity of your feelings resulting from not being able to smoke a traditional cigarette and not being able to use a heated tobacco product.

1. They were stronger when I smoked traditional cigarettes
2. They are stronger when I use heated tobacco products
3. Hard to say
4. They are equally strong
5. They are equally negligible

Q31. Did smoking traditional cigarettes affect your lifestyle? If so, what aspects did it affect? You can select more than one answer.

Q31_01 - It did not affect my lifestyle

Q31_02 - Diet

Q31_03 - Choice of friends

Q31_04 - Life plans

Q31_05 - Family relationships

Q31_06 - Relaxation and leisure time

Q31_07 - Habits

Q31_08 - Places visited

Q31_09 - Daily schedule

Q31_10 - Sports and recreation

Q31_11 - Other

Q32. Does using heated tobacco products affect your lifestyle? If so, what aspects does it affect? You can select more than one answer.

Q32_01 - It does not affect my lifestyle

Q32_02 - Diet

Q32_03 - Choice of friends

Q32_04 - Life plans

Q32_05 - Family relationships

Q32_06 - Relaxation and leisure time

Q32_07 - Habits

Q32_08 - Places visited

Q32_09 - Daily schedule

Q32_10 - Sports and recreation

Q32_11 - Other

Q33. Compare the impact of smoking traditional cigarettes and using heated tobacco products on your lifestyle.

1. It was stronger when I smoked traditional cigarettes
2. It is stronger when I use heated tobacco products
3. Hard to say
4. They are equally strong
5. They are equally negligible

Q34. Was smoking traditional cigarettes an expression of your attitude? If so, what attitude? You can select more than one answer.

Q34_01 - There was no specific attitude behind it

Q34_02 - Attitude of protest

Q34_03 - Demonstration of maturity

Q34_04 - Rebellion against rules

Q34_05 - Fashion

Q34_06 - Seeking new experiences

Q34_07 - The sense of being in control

Q34_08 - Manifestation of views

Q34_09 - To be taken seriously

Q34_10 - Desire to resemble important people to me

Q34_11 - Other

Q35. Is using heated tobacco products an expression of your attitude? If so, what attitude? You can select more than one answer.

Q35_01 - There is no specific attitude behind it

Q35_02 - Attitude of protest

Q35_03 - Demonstration of maturity

Q35_04 - Rebellion against rules

Q35_05 - Fashion

Q35_06 - Seeking new experiences

Q35_07 - The sense of being in control

Q35_08 - Manifestation of views

Q35_09 - To be taken seriously

Q35_10 - Desire to resemble important people to me

Q35_11 - Other

Q36. Compare your commitment to attitudes associated with smoking traditional cigarettes and attitudes associated with using heated tobacco products.

1. It was greater when I smoked traditional cigarettes
2. It is stronger when I use heated tobacco products
3. Hard to say
4. It was and is equally strong
5. It was and is equally negligible

Q37. Did you feel pressure against smoking traditional cigarettes?

1. Very often
2. Often
3. Rarely
4. Very rarely
5. I did not feel it

Q38. Do you feel pressure against using heated tobacco products?

1. Very often
2. Often
3. Rarely
4. Very rarely
5. I do not feel it

Q39. Compare the scale of social pressure against smoking traditional cigarettes and using heated tobacco products.

1. It was stronger when I smoked traditional cigarettes
2. It is stronger when I use heated tobacco products
3. Hard to say
4. They are equally strong
5. They are equally negligible
6. I do not feel pressure

Q40. In the case of smoking traditional cigarettes, what type of pressure did you find the most severe? You can select more than one answer.

Q40_01 - Designated smoking areas

Q40_02 - Family criticism

Q40_03 - Criticism from friends

Q40_04 - Labels on the packs

Q40_05 - Social stigma

Q40_06 - Other

Q41. In the case of using heated tobacco products, what type of pressure do you find the most severe?

Q41_01 - Designated smoking areas

Q41_02 - Family criticism

Q41_03 - Criticism from friends

Q41_04 - Labels on the packs

Q41_05 - Social stigma

Q41_06 - Other

Q42. Compare the severity of social pressure against smoking traditional cigarettes and using heated tobacco products.

1. It was more severe when I smoked traditional cigarettes
2. It is more severe when I use heated tobacco products
3. Hard to say
4. They are equally severe
5. They are equally negligible

Q43. What impact did social pressure against smoking traditional cigarettes have on you?

1. It had such a big impact that I decided to switch to heated tobacco products
2. It had a moderate impact, but it made me constantly think about quitting
3. It had a small impact
4. It had no impact

Q44. What impact does social pressure against using heated tobacco products have on you?

1. It has such a big impact that I want to attempt quitting heated tobacco products
2. It has a moderate impact, but it makes me constantly think about quitting
3. It has a small impact
4. It has no impact

Q45. Compare the effectiveness of social pressure against smoking traditional cigarettes and using heated tobacco products.

1. It was more effective when I smoked traditional cigarettes
2. It is more effective when I use heated tobacco products
3. Hard to say
4. They are equally effective
5. They are equally insignificant

Q46. When smoking traditional cigarettes, did you feel a strong need to smoke?

1. Definitely yes
2. Probably yes
3. I have no opinion
4. Probably not
5. Definitely not

Q47. Do you feel a strong need to use heated tobacco products?

1. Definitely yes
2. Probably yes
3. I have no opinion
4. Probably not
5. Definitely not

Q48. Compare which need was stronger.

1. It was stronger when I smoked traditional cigarettes
2. It is stronger when I use heated tobacco products
3. They are equally strong
4. Hard to say
5. I don't feel addicted

Q49. What bothered you when you smoked traditional cigarettes? You can select more than one answer.

Q49_01 - Nothing bothered me

Q49_02 - The smell of smoke

Q49_03 - The smell on clothes

Q49_04 - Fear of illness

Q49_05 - Urging to quit

Q49_06 - Harming others

Q49_07 - Social stigma

Q49_08 - Bad breath

Q49_09 - Other

Q50. What bothers you when you use heated tobacco products? You can select more than one answer.

Q50_01 - Nothing bothers me

Q50_02 - The smell of the product

Q50_03 - The smell on clothes

Q50_04 - Fear of illness

Q50_05 - Urging to quit

Q50_06 - Harming others

Q50_07 - Social stigma

Q50_08 - Bad breath

Q50_09 - Other

Q51. Compare the level of inconvenience between smoking traditional cigarettes and using heated tobacco products.

1. It was greater when I smoked traditional cigarettes
2. It is greater when I use heated tobacco products
3. Hard to say
4. It is similar
5. They are equally negligible

Q52. What motivated you to switch to heated tobacco products? You can select more than one answer.

Q52_01 - Fear of illness

Q52_02 - Desire to reduce smoking

Q52_03 - Hope for changing habits

Q52_04 - Loss of fitness

Q52_05 - Feeling unwell

Q52_06 - Pressure from family

Q52_07 - Personal decision

Q52_08 - Influence of others

Q52_09 - Cost

Q52_10 - Other

Q53. What would motivate you to stop using heated tobacco products? You can select more than one answer.

Q53_01 - Fear of illness

Q53_02 - Desire to reduce smoking

Q53_03 - Hope for changing habits

Q53_04 - Loss of fitness

Q53_05 - Feeling unwell

Q53_06 - Pressure from family

Q53_07 - Personal decision

Q53_08 - Influence of others

Q53_09 - Cost

Q53_10 - Other

Q54. How do you assess your decision to switch to heated tobacco products?

1. Beneficial mainly for my physical health
2. Beneficial mainly for my mental health
3. Beneficial mainly for my overall well-being
4. Beneficial mainly for my relationships with others
5. I have no opinion
6. Not beneficial

Q55. What does switching from traditional cigarettes to heated tobacco products primarily represent for you?

1. It is a conscious step towards quitting the habit
2. It is part of a broader plan to quit the habit and change my lifestyle
3. It is a hope to increase my motivation to quit the habit
4. It is an attempt to change habits
5. It is just a change of product and nothing more

Q56. What has switching to heated tobacco products primarily changed for you?

1. It motivated me to decide to quit the habit
2. It motivated me to make broader lifestyle changes
3. Switching was the last resort to quit smoking
4. I feel a greater quality of life today
5. It didn't change anything

Q57. Do you think about returning to smoking traditional cigarettes?

1. I will definitely not return to smoking traditional cigarettes
2. I will probably not return to smoking traditional cigarettes
3. I don't know, it's hard to say
4. I might return to smoking
5. I will definitely return to smoking

Q58. While smoking traditional cigarettes, did you simultaneously use other substances? You can select more than one answer.

Q58_01 - I did not use any

Q58_02 - Coffee

Q58_03 - Alcohol

Q58_04 - Energy drinks

Q58_05 - Drugs

Q58_06 - Other intoxicants

Q58_07 - Other

Q59. While using heated tobacco products, do you simultaneously use other substances?

Q59_01 - I do not use any

Q59_02 - Coffee

Q59_03 - Alcohol

Q59_04 - Energy drinks

Q59_05 - Drugs

Q59_06 - Other intoxicants

Q59_07 - Other

Q60. Please compare your consumption of other substances.

1. It was greater when I smoked traditional cigarettes
2. It is greater when I use heated tobacco products
3. Hard to say
4. They are equally high
5. They are equally negligible

Q61. When you smoked traditional cigarettes, how did you usually dispose of cigarette butts?

1. Always in the trash or ashtray
2. Usually in the trash or ashtray
3. Anywhere
4. In the packaging
5. I don’t remember

Q62. How do you dispose of waste from heated tobacco products?

1. Always in the trash or ashtray
2. Usually in the trash or ashtray
3. Anywhere
4. In the packaging
5. Hard to say

Q63. Compare the ability to control waste disposal.

1. It was easier when I smoked traditional cigarettes
2. It is easier when I use heated tobacco products
3. Hard to say
4. I control it equally well
5. I never control it

Q64. In terms of health hazards, do you believe that:

1. Traditional cigarettes are more harmful
2. Heated tobacco products are more harmful
3. Both products are equally harmful
4. I have no knowledge on this topic

Q65. Where do you get your beliefs about the harmfulness of traditional cigarettes and heated tobacco products?

Q65_01 - From the Internet

Q65_02 - From informational brochures by government institutions

Q65_03 - From friends

Q65_04 - From statements by influencers, bloggers, etc.

Q65_05 - From general information on social media (e.g., Facebook, Twitter, Instagram, etc.)

Q65_06 - From channels on online platforms (e.g., YouTube, TikTok, etc.)

Q65_07 - From radio and television

Q65_08 - From a doctor

Q65_09 - From healthcare workers who are not doctors

Q65_10 - I don’t think about it

Q66. Do you feel well-informed about the harmfulness of traditional cigarettes and heated tobacco products?

1. No, but I don’t feel the need to be
2. No, but I don’t know where to find such information
3. Yes, I know everything I want to know
4. Yes, but I would like to have the option to verify my knowledge further

Q67. Do you think that government information on the scale of harmfulness of traditional cigarettes compared to heated tobacco products is sufficient?

1. It is easily accessible and sufficient
2. It is easily accessible, but insufficient
3. It is hard to access, but sufficient
4. It is hard to access and insufficient

**Statistical analysis**


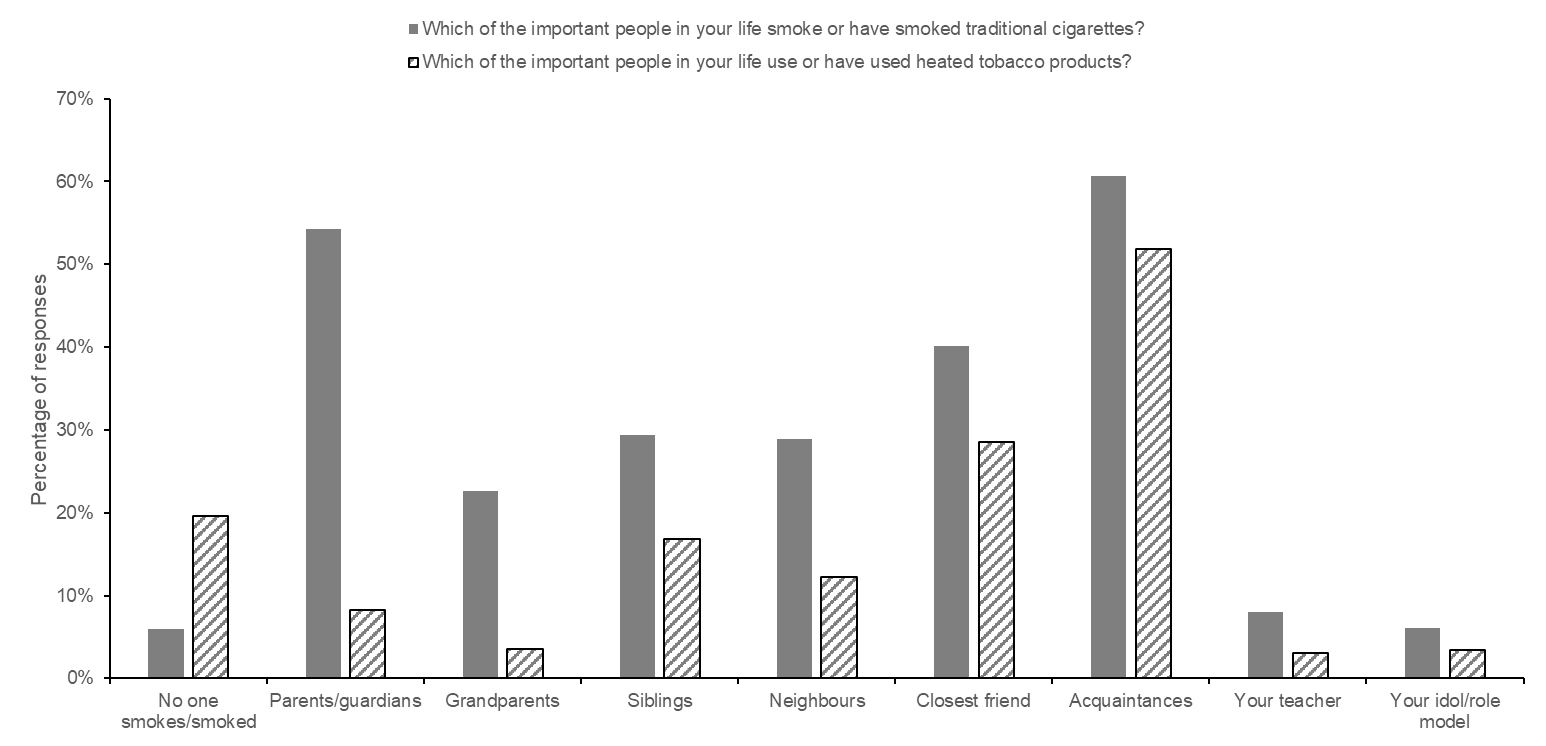


Figure S1. Comparison of which important people, according to respondents, smoked Traditional Cigarettes or used Heated Tobacco Products.


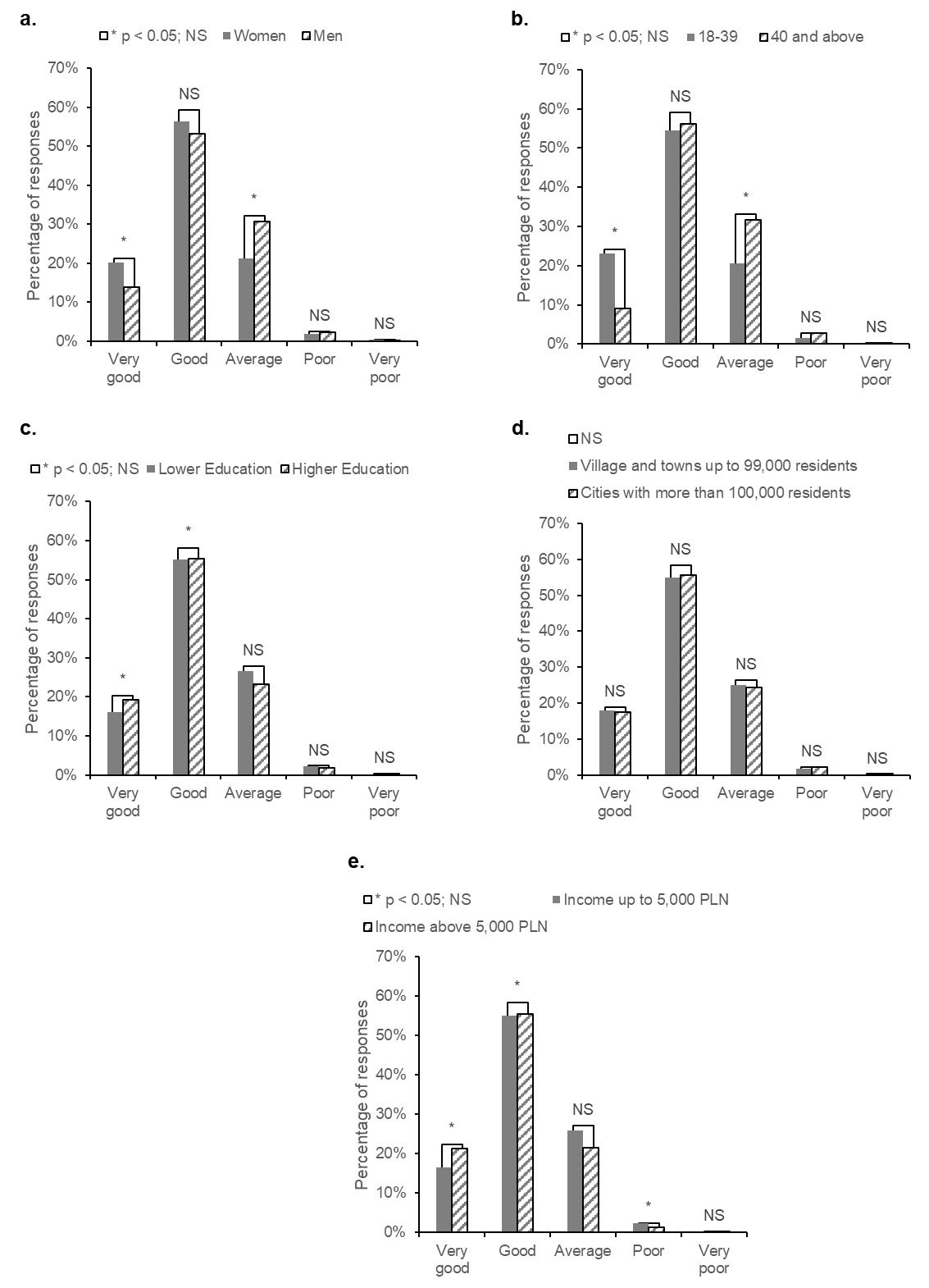


Figure S2. Assessment of respondents’ reported general health status. Adjustment for gender (a), age (b), education (c), place of residence (d), income (e).

Abbreviations: p, *P* value; NS, non-significant


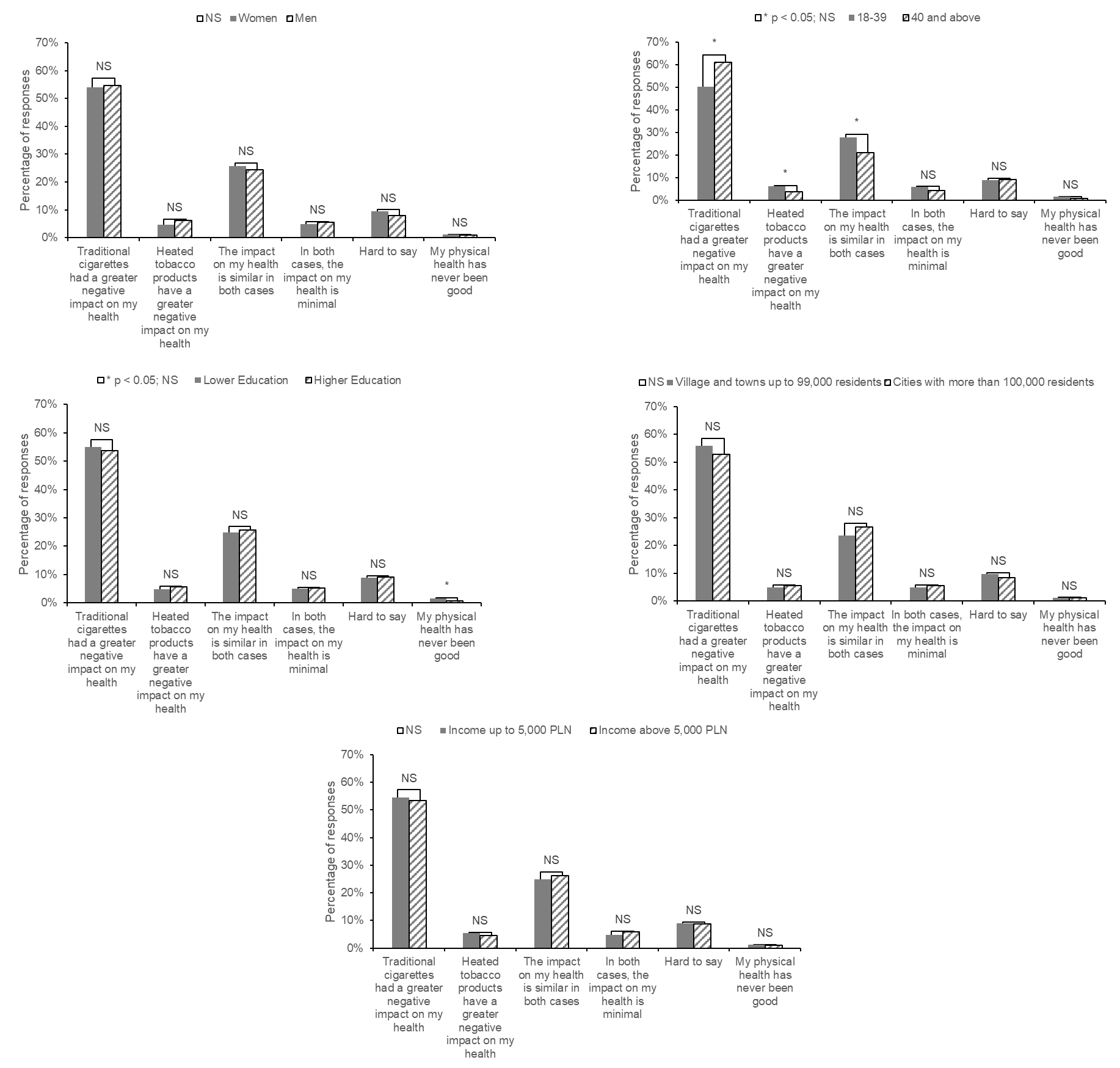


Figure S3. Comparison of the impact of Traditional Cigarettes and Heated Tobacco Products on respondents’ reported physical health. Adjustment for gender (a), age (b), education (c), place of residence (d), income (e).

Abbreviations: p, *P* value; NS, non-significant


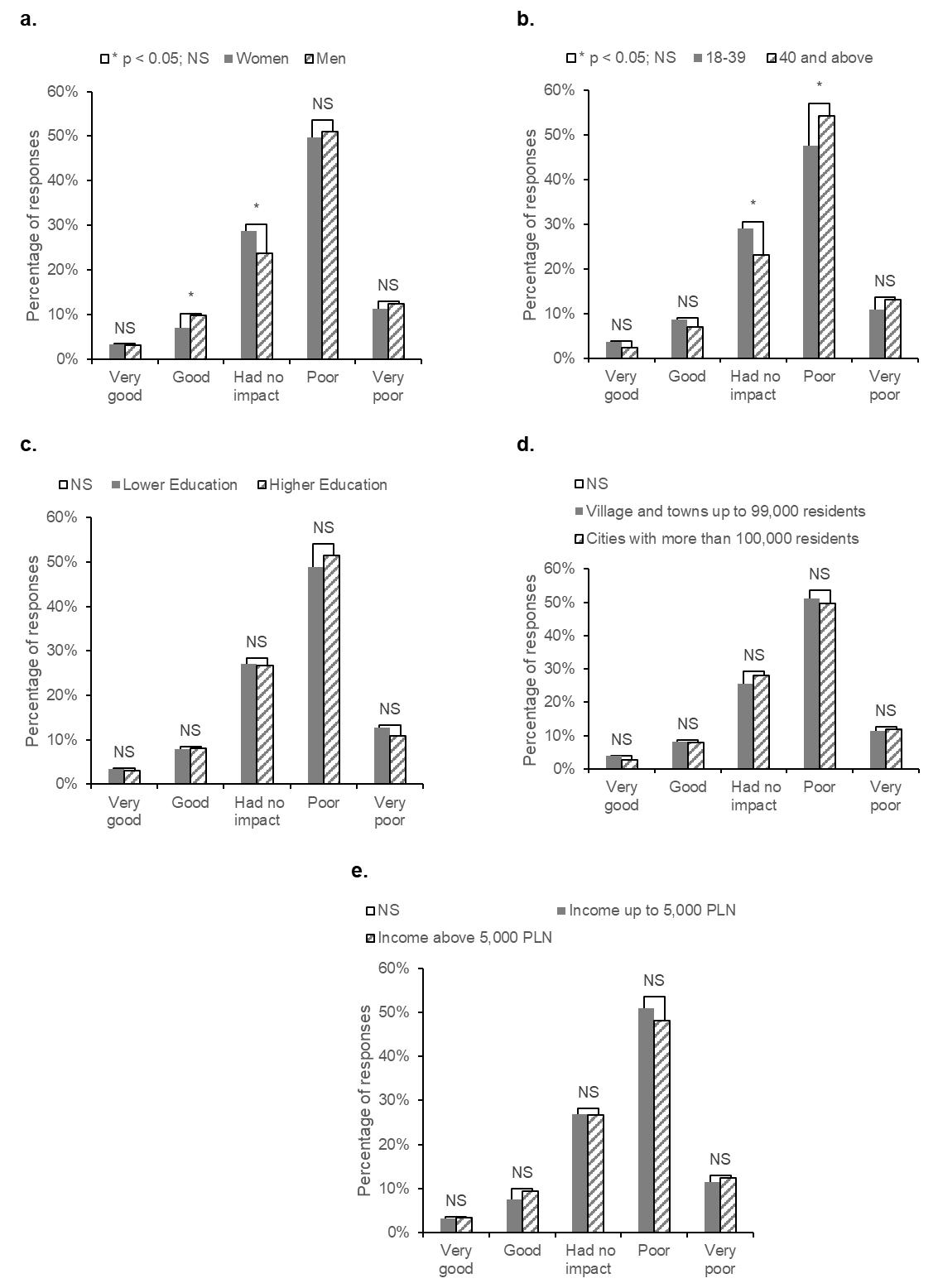


Figure S4. Comparison of the Impact of Traditional Cigarettes on respondents’ reported physical condition. Adjustment for gender (a), age (b), education (c), place of residence (d), income (e).

Abbreviations: p, *P* value; NS, non-significant


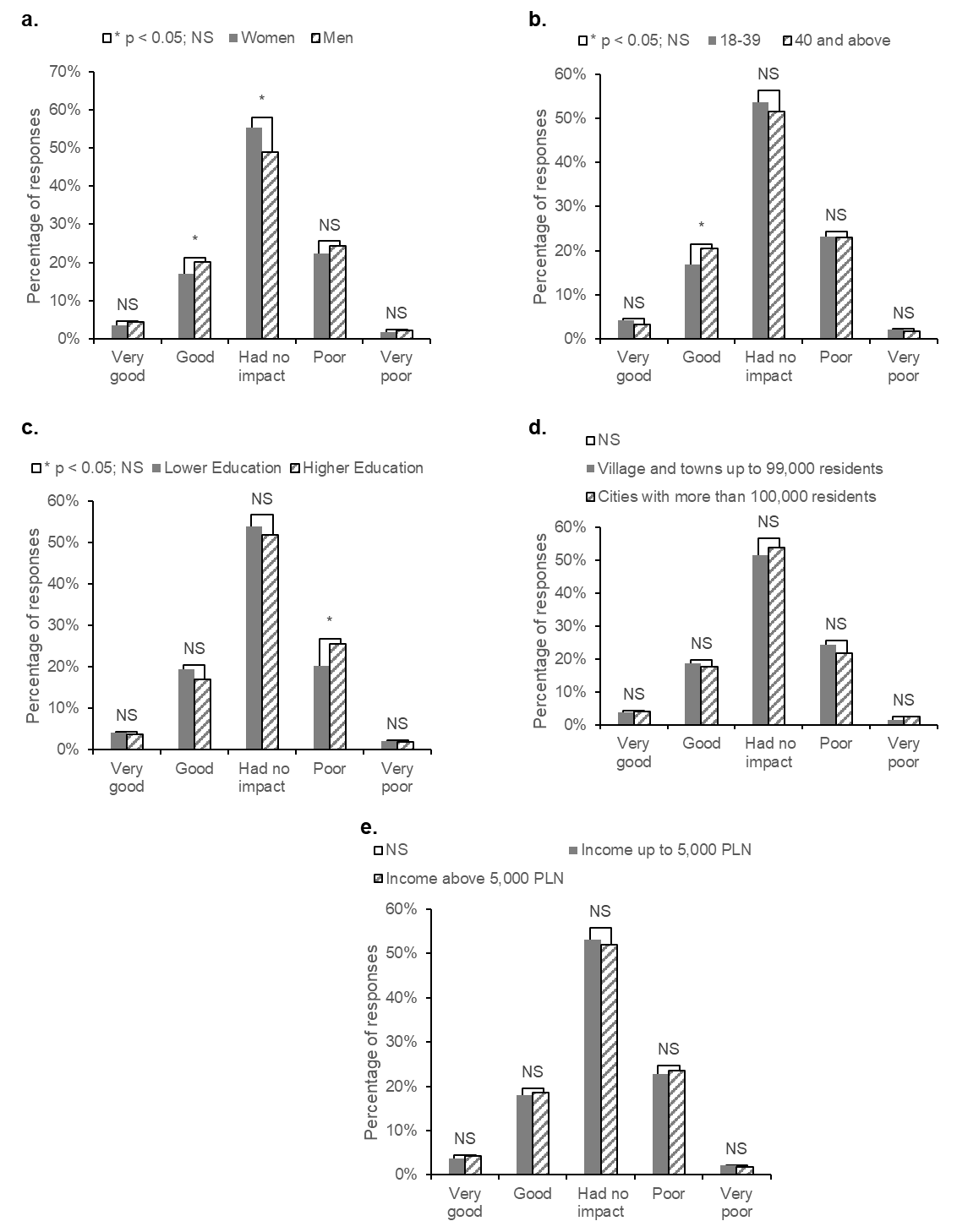


Figure S5. Comparison of the Impact of Heated Tobacco Products on respondents’ reported physical condition. Adjustment for gender (a), age (b), education (c), place of residence (d), income (e).

Abbreviations: p, *P* value; NS, non-significant


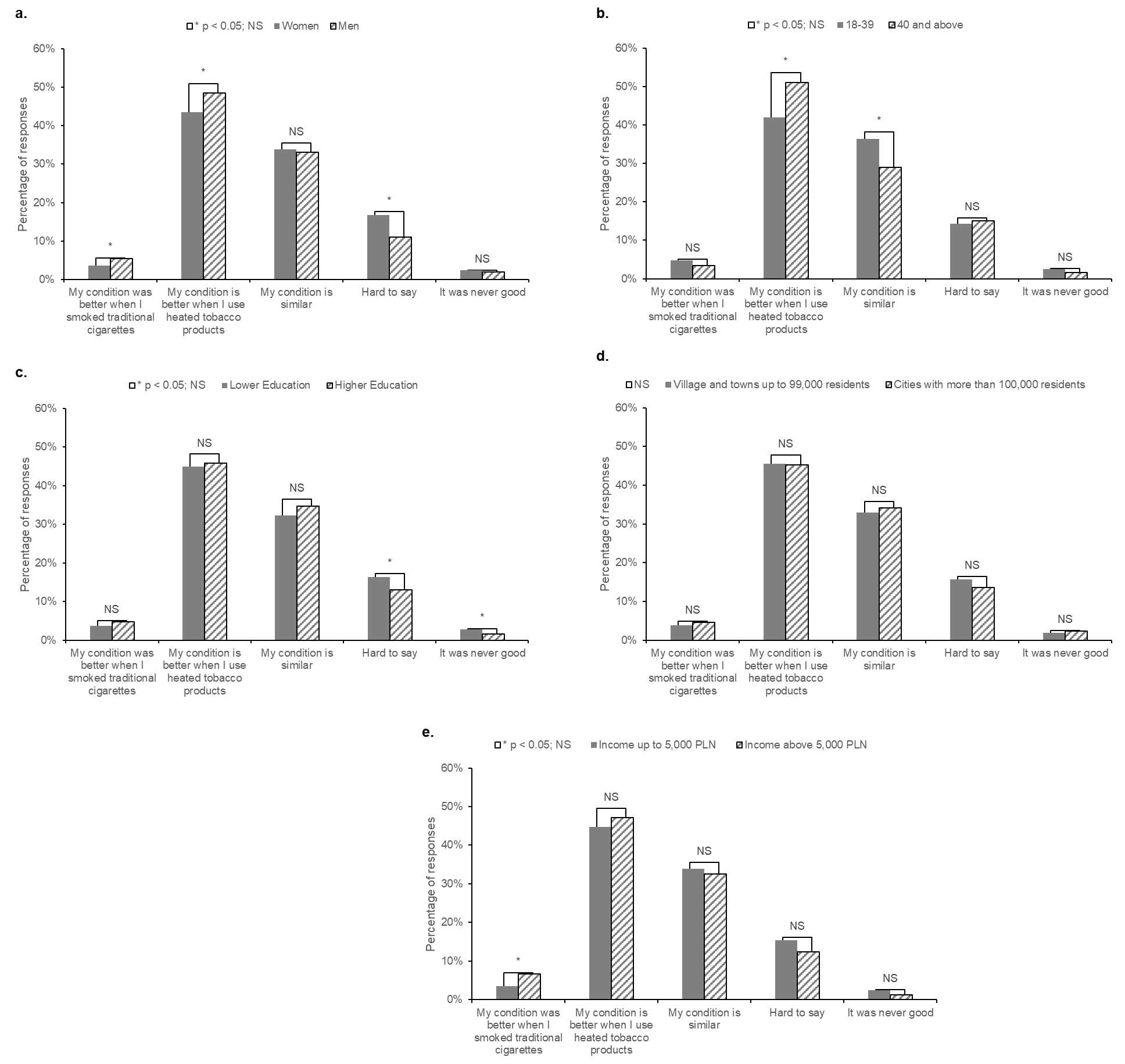


Figure S6. Comparison of the impact of Traditional Cigarettes and Heated Tobacco Products on respondents’ reported physical condition. Adjustment for gender (a), age (b), education (c), place of residence (d), income (e).

Abbreviations: p, *P* value; NS, non-significant

**A multinomial logistic regression model was constructed to assess the effects of the predictors—sex, age, education, place of residence, and income—on respondents’ evaluation of the impact of smoking traditional cigarettes or using heated tobacco products on their physical health (Question Q15). In this model, response categories 1, 2, 3, and 4 were treated as dependent outcome categories, whereas category 5 served as the reference group. Category 6 was excluded because it did not contribute substantively to the model. The multinomial logistic regression model demonstrated acceptable overall fit, as confirmed by a statistically significant model test (χ²[25] = 58.6; p < .00016). However, the goodness-of-fit indices indicated a relatively low explanatory power (AIC = 6277; McFadden’s R² = 0.00934). Omnibus likelihood ratio tests showed that several predictors included in the model were statistically significant (p < 0.05).**

Table S2. Multinomial logistic regression model assessing the effects of predictors—sex, age, education, place of residence, and income—on respondents’ evaluation of the impact of smoking traditional cigarettes or using heated tobacco products on physical health.

**A multinomial logistic regression model was constructed to assess the effects of the predictors—sex, age, education, place of residence, and income—on respondents’ evaluation of the impact of smoking traditional cigarettes or using heated tobacco products on their physical condition (Question Q12). In this model, response categories 1, 2, and 3 were treated as dependent outcome categories, whereas category 4 served as the reference group. Category 5 was excluded because it did not contribute substantively to the model. The multinomial logistic regression model demonstrated overall fit to the data, as confirmed by a statistically significant model test (χ²[20] = 72.7; p < .00001). However, the goodness-of-fit indices indicated a relatively low explanatory power (AIC = 6093; McFadden’s R² = 0.0119). Omnibus likelihood ratio tests showed that several predictors included in the model were statistically significant (p < 0.05).**

**Table S3. Multinomial logistic regression model assessing the effects of predictors—sex, age, education, place of residence, and income—on respondents’ evaluation of the impact of smoking traditional cigarettes or using heated tobacco products on physical condition.**
